# Severe necrosis of the glans penis associated with calciphylaxis treated by partial penectomy

**DOI:** 10.1002/iju5.12166

**Published:** 2020-05-26

**Authors:** Masato Tezuka, Hiroya Mizusawa, Manabu Tsukada, Yuji Mimura, Takaaki Shimizu, Aya Kobayashi, Yasufumi Takahashi, Toshitaka Maejima

**Affiliations:** ^1^ Department of Urology National Hospital Organization Shinshu Ueda Medical Center Ueda Nagano Japan; ^2^ Department of Dermatology National Hospital Organization Shinshu Ueda Medical Center Ueda Nagano Japan; ^3^ Department of Nephrology National Hospital Organization Shinshu Ueda Medical Center Ueda Nagano Japan; ^4^ Department of Pathology and Laboratory Medicine National Hospital Organization Shinshu Ueda Medical Center Ueda Nagano Japan

**Keywords:** calcification, diabetic nephropathy, dialysis, penile necrosis, penile pain

## Abstract

**Introduction:**

Calciphylaxis is characterized by marked vascular calcification and painful skin ulcers, and it has a poor prognosis.

**Case presentation:**

The patient was a 72‐year‐old male. He was referred for penile pain. He had a 4‐year history of dialysis therapy under a diagnosis of diabetic nephropathy. Black and yellow necrosis was observed involving the entire glans, accompanying severe pain. Computed tomography revealed marked calcification involving the thoracoabdominal aorta to iliac arteries, the dorsal artery of the penis and the corpus cavernosum, leading to a diagnosis of calciphylaxis. Penile pain gradually exacerbated and partial penectomy was performed. After surgery, penile pain promptly subsided. Pathological examination confirmed marked calcification of the microvascular wall and narrowing of the lumen.

**Conclusion:**

We reviewed 15 Japanese patients with calciphylaxis who had undergone penile surgery. Surgical treatment was considered to be effective at relieving penile pain, but the prognosis remained poor.

**Abbreviations & Acronyms**
Ca: calcium
CRP: C‐reactive protein
Hb: hemoglobin
HbA_1c_: hemoglobin A_1c_
IP: inorganic phosphorus
I‐PTH: intact parathyroid hormone
PLT: platelet
WBC: white blood cell

**Keynote message**

Partial penectomy was performed on a patient with diabetic nephropathy for severe calciphylaxis‐related penile pain. After surgery, penile pain promptly subsided. In the reviewed 15 Japanese patients with calciphylaxis, penile surgery was considered to be effective at relieving penile pain, but the prognosis remained poor.

## Introduction

Calciphylaxis is a refractory disease characterized by marked vascular calcification and painful skin ulcers that primarily develop in dialysis patients, and it has a poor prognosis. It frequently develops in the limbs/trunk/fingers/toes.^1^, ^2^ We report a patient with calciphylaxis‐related necrosis of the penis undergoing dialysis therapy for diabetic nephropathy in whom partial penectomy reduced severe penile pain.

## Case presentation

The patient was a 72‐year‐old male. He was referred for penile pain. He had a 4‐year history of dialysis therapy under a diagnosis of diabetes mellitus, which had been made 11 years previously. No urine production was observed, and 4‐hour hemodialysis was performed three times a week. He also had a history of acute myocardial infarction, lacunar infarction, hypothyroidism and diabetic retinal detachment. Insulin glargine, aspirin, furosemide, amezinium metilsulfate, levothyroxine sodium hydrate and allopurinol were administered for the respective diseases.

Pain of the glans had persisted for 10 days, but the patient was unable to confirm the site of pain due to blindness. On consultation at the previous hospital, necrosis of the glans was observed and he was referred to our department for treatment.

His height, body weight, blood pressure, pulse rate, and body temperature were 160 cm, 64 kg, 142/74 mmHg, 77/min, and 36.8°C, respectively.

**Fig. 1:**
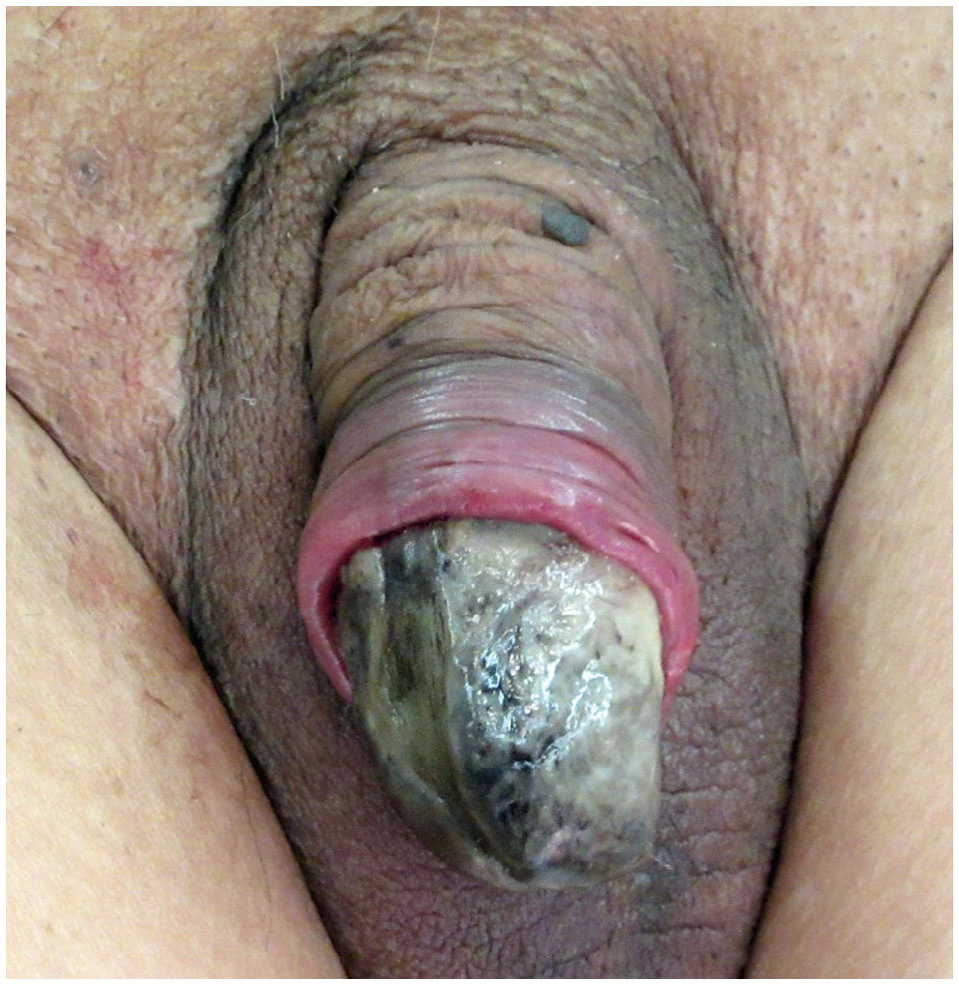
Glans with severe pain. Necrosis‐related discoloration was noted.

Yellow and black necrosis of the entire glans with severe pain was noted (Fig. 1), and purulent discharge with odor was observed. Ulcers of the bilateral lower limbs and right dorsal hand with irregular yellow necrosis, dark red spots, and black keratotic nodules were noted.

Laboratory examination on admission demonstrated corrected Ca, IP, I‐PTH, CRP, HbA_1c_, WBC, Hb, and PLT levels of  9.7 mg/dL,    7.4 mg/dL, 97 pg/mL, 11.5 mg/dL, 7.3%,  13 500/μL, 18.3 g/dL and 23.4 × 10^4^/μL, respectively.

**Fig. 2:**
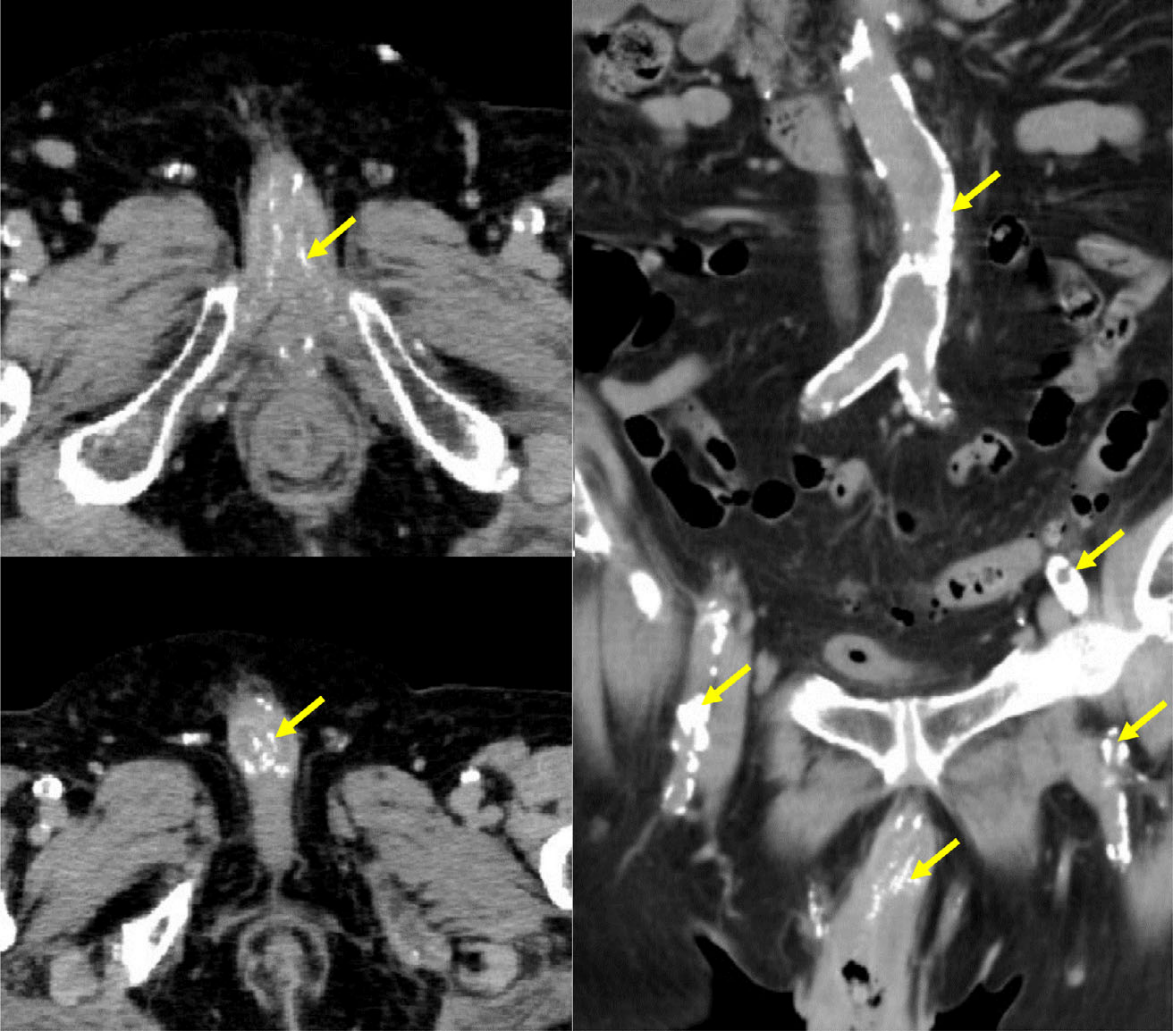
Plain computed tomography. Marked vascular calcification was observed. Aorta, iliac artery and penis blood vessel (arrows).

Plain computed tomography of the thorax and abdomen revealed marked calcification involving the thoracoabdominal aorta to external/internal iliac arteries. Calcification of the dorsal artery of the penis and ectopic calcification of the corpus cavernosum were also observed (Fig. 2). On urethroscopy, urethral stricture in the anterior urethra was noted, but the investigation was insufficient because it induced further penile pain. Blood flow examination in the penis using color Doppler ultrasound was not performed.

After admission, lavage and debridement were performed based on a diagnosis of calciphylaxis or necrosis related to intravascular thrombus at the department of dermatology. However, pain control of the glans was poor and there was no reduction of necrosis. Partial penectomy was performed 8 days after admission. The glans was dissected at an area proximal to the coronary sulcus. There was no hemorrhage. The external urethral meatus was created by a standard procedure. After surgery, penile pain promptly subsided. Paleness in a portion of the surgical wound, purulent discharge and fever were observed, but perineal care, debridement and antimicrobial drug administration were carried out.

**Fig. 3:**
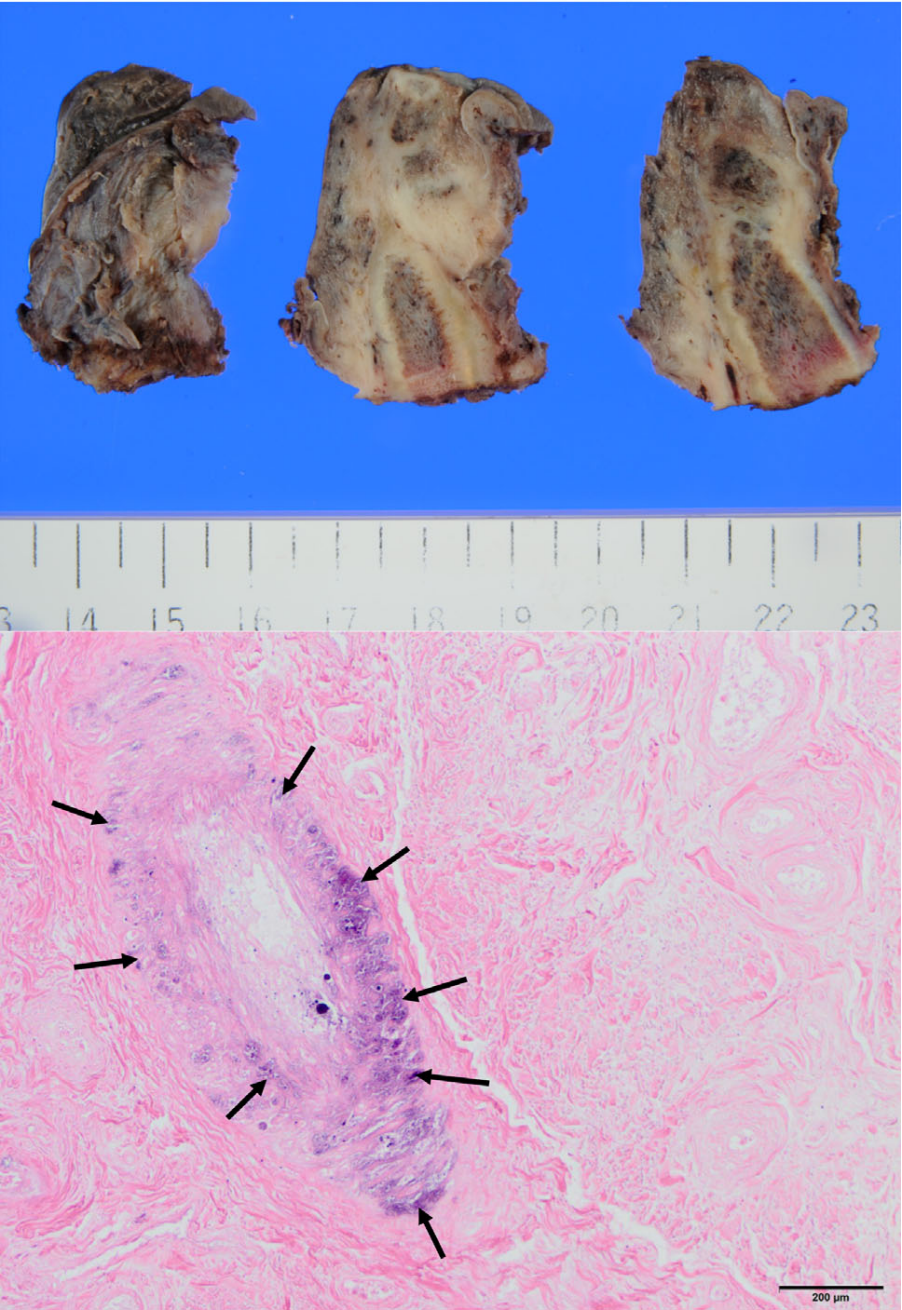
Pathological findings of the resected specimen: macroscopic (upper) and microscopic (lower) findings. Marked calcification in the media (arrows), thickening of the intima and narrowing of the lumen in the small artery were observed. Hematoxylin and eosin staining.

Pathological macroscopic findings included ulceration, necrosis and abscess formation. Microscopy demonstrated marked calcification of the media in the small arteries, and thickening of the intima and lumen stenosis was noted, suggesting calciphylaxis (Fig. 3).

The patient was transferred to his previous dialysis hospital 34 days postoperatively, with improvement in his general condition. The course had been favorable based on postoperative follow‐up, but he died of pneumonia 11 months after surgery.

## Discussion

Calciphylaxis primarily develops in dialysis patients. It is characterized by purpura/induration of the skin and severe pain, rapidly causing ulcers and necrosis. Histopathologically, ischemia associated with arterial stenosis related to marked calcification of small arteries and soft tissue is primarily observed. This condition is considered to be different from arteriosclerosis, which is generally defined as atherosclerosis related to hypertension, diabetes mellitus or dyslipidemia.^1^ The pathogenesis remains to be clarified.

The annual incidence of calciphylaxis in Europe and the United States is 3.5 per 1000 patients chronically receiving dialysis.^3^ In Japan, it is ≤0.1 per 1000 patients, being markedly lower. However, one study suggested that this disease is not sufficiently recognized.^4^ Doppler ultrasound examination is necessary to evaluate penile blood flow in a patient whose general condition is fair.

It frequently develops in the lower limbs/trunk. Calciphylaxis of the penis accounts for approximately 3%.^2^ The prognosis is poor, and the mortality rate within 1 year ranges from 45 to 80%. The most frequent cause of death is sepsis.^1^

No treatment has been established. In addition to conservative treatment, surgery is sometimes performed to ameliorate pain or remove the source of infection, but few case reports have been published.

In Japan, 14 patients treated by surgery for calciphylaxis‐related necrosis of the penis have been reported. We reviewed 15 patients, including ours (Table 1). Hayashi *et al*.^4^ proposed diagnostic criteria for calciphylaxis consisting of clinical symptoms and pathological findings of the skin. Among reports on necrosis of the penis, patients meeting the diagnostic criteria were added to the above group. Concerning age, a peak was reached in the latter half of the 50s. Regarding clinical symptoms, penile pain was noted in all patients and most complained of severe pain. Furthermore, erosion/ulcers of the glans and skin discoloration were observed in all patients. Necrosis/ulcer of the lower limbs was noted in ≥70%. All patients had received dialysis. In 50% of the patients, the duration of dialysis was ≥5 years, but it was <1 year in two patients. In 87%, a diagnosis of diabetes mellitus had been made, but blood glucose control was favorable in some. Regarding techniques, partial penectomy was performed on approximately 80%. The disappearance or reduction of postoperative penile pain was achieved in eight of nine patients for whom such pain was described. Surgery may have had specific effects on calciphylaxis‐related penile pain. Concerning the outcome, the longest follow‐up period among the surviving patients was 8 months. No study has reported the long‐term outcome. Of six patients who died, four died within 3 months. The prognosis of surgically treated patients was markedly poor, as previously reported.

**Table 1:** Clinical findings for previously reported Japanese patients with calciphylaxis treated by penile surgery

	*n* = 15
Age	59 (41–73)
Symptom	
Penile pain	15
Necrosis of the glans	15
Necrosis of the lower limbs	10/14
Diabetes mellitus	
Present	13
Absent	2
Duration of dialysis	
<5 years	7/14
≥5 years	7/14
Technique	
Partial penectomy	11
Total amputation	4
Postoperative pain	
Disapperance	3/9
Improvement	5/9
No change	1/9
Outcome	
Survival	
<1 year	5/12
≥1 year	0/12
Death	
<1 year	6/12
≥1 year	1/12

## Conclusion

We reported a patient in whom partial penectomy for calciphylaxis‐related necrosis of the penis reduced pain. Based on a review of 15 Japanese patients with calciphylaxis treated by penile surgery, surgery was considered to be effective at relieving penile pain due to calciphylaxis, but the prognosis was poor.

## Conflict of interest

The authors declare no conflict of interest.
